# Detection of UDP-Based Volumetric DDoS Attacks in IoT Environments Using LSTM with Temporal Attention Mechanism

**DOI:** 10.3390/s26134237

**Published:** 2026-07-03

**Authors:** Bengisu Eda Aydin, Zafer Güney, Hakan Aydin

**Affiliations:** 1 Department of Artificial Intelligence Engineering, Institute of Graduate Studies, Istanbul Topkapi University, Istanbul 34310, Turkey; bengisuedaaydin@stu.topkapi.edu.tr; 2 Department of Computer Engineering, Faculty of Engineering and Architecture, Istanbul Topkapi University, Istanbul 34310, Turkey; zaferguney@topkapi.edu.tr

**Keywords:** IoT security, DDoS detection, UDP Flood attack, Long Short-Term Memory (LSTM), temporal attention mechanism

## Abstract

Internet of Things (IoT) environments, similarly to traditional network infrastructures, are highly vulnerable to volumetric Distributed Denial of Service (DDoS) attacks. Detecting such attacks remains challenging due to their bursty and short-lived nature, particularly in User Datagram Protocol (UDP) flood traffic, which often blends into normal traffic fluctuations. Conventional deep learning (DL) approaches, particularly Long Short-Term Memory (LSTM) networks, assign uniform importance to all time steps, limiting their ability to capture temporally localized burst patterns critical for identifying UDP-based volumetric attacks. To address this limitation, this study proposes LSTM-IoT, an attention-enhanced intrusion detection framework that integrates a temporal attention mechanism into an LSTM architecture. The model selectively emphasizes informative time intervals while suppressing irrelevant temporal segments, improving discrimination between benign and attack traffic. Evaluated on UDP traffic flows from the CICDDoS2019 dataset, LSTM-IoT achieves a detection accuracy of 99.93%, outperforming a baseline LSTM model. The results confirm that the proposed DL-based model effectively detects UDP-based volumetric DDoS attacks in IoT environments.

## 1. Introduction

The Confidentiality, Integrity, and Availability (CIA) triad defines the core security objectives of modern networked systems, while Authentication, Authorization, and Accounting (AAA) mechanisms provide controlled and accountable access in distributed environments [1,2]. As cyberspace becomes increasingly interconnected, ensuring resilience against large-scale cyber threats has become a fundamental requirement [3]. Among these threats, Distributed Denial of Service (DDoS) attacks remain a major challenge to network availability, particularly in cloud and Internet of Things (IoT) environments. Volumetric DDoS attacks are specifically designed to exhaust bandwidth resources through high-rate traffic generation, resulting in service degradation or complete service disruption. Within this class, User Datagram Protocol (UDP)-based flooding attacks are particularly effective due to the connectionless nature of the UDP, which enables high-volume traffic generation with minimal overhead and limited traceability.

IoT environments further exacerbate this vulnerability due to their large-scale deployment, heterogeneous architecture, and severe resource constraints. Limited computational and memory capabilities significantly reduce the ability of IoT devices to withstand sudden traffic surges, making them highly susceptible to volumetric DDoS attacks even under partial node compromise. To mitigate these challenges, deep learning (DL)-based intrusion detection systems (IDS) have been extensively investigated. In particular, Long Short-Term Memory (LSTM) networks are widely adopted for modeling temporal dependencies in network traffic. However, existing approaches largely treat DDoS detection as a generic classification problem and fail to explicitly capture the bursty and short-duration characteristics of UDP-based volumetric attacks. Moreover, conventional LSTM architectures assign uniform importance to all time steps, which limits their ability to detect temporally localized traffic bursts embedded within normal traffic fluctuations.

To address these limitations, this study proposes LSTM-IoT, a lightweight intrusion detection framework that integrates a temporal attention mechanism into an LSTM architecture for detecting UDP-based volumetric DDoS attacks in IoT environments. The proposed mechanism adaptively focuses on informative temporal intervals associated with abrupt traffic variations while suppressing irrelevant segments, thereby improving discrimination between benign and attack traffic under resource-constrained conditions.

The contributions of this study to the literature can be expressed as follows:

- A temporal attention-enhanced LSTM framework (LSTM-IoT) is proposed for burst-aware detection of UDP-based volumetric DDoS attacks.
- A computationally efficient attention mechanism is introduced to enhance detection capability with minimal parameter overhead.
- Extensive experiments on the CICDDoS2019 dataset demonstrate that the proposed method achieves 99.93% accuracy, outperforming a baseline LSTM model while maintaining computational efficiency.

The remainder of this paper is organized as follows. Section 2 presents the materials and methods, Section 3 provides the literature review, Section 4 gives the background, Section 5 describes the proposed LSTM-IoT system, Section 6 discusses the findings, and Section 7 concludes the study.

## 2. Materials and Methods

This study uses the publicly available CICDDoS2019 dataset [4], which contains benign and malicious network traffic records, including multiple DDoS attack types such as UDP Flood, SYN Flood, DNS Amplification, LDAP, NetBIOS, NTP, MSSQL, UDP-Lag, PortMap, and SNMP. The dataset comprises approximately 50 million traffic samples with around 57,000 benign samples. For this study, only UDP-based traffic flows are selected to enable focused analysis of UDP Flood volumetric DDoS attacks. Raw data is preprocessed by removing duplicate and irrelevant records, handling missing values via mean imputation, encoding labels for binary classification (benign = 0, attack = 1), and applying min-max normalization to scale features to the range [0, 1]. Key features selected for model training include packet counts, byte statistics, flow duration, port information, IP addresses, and forward packet length. The preprocessed dataset is split into training (70%), validation (15%), and testing (15%) sets.

The proposed LSTM-IoT framework is based on an LSTM network enhanced with a temporal attention mechanism. Standard LSTM models process all time steps with equal importance, which limits their ability to detect short-duration, high-magnitude traffic bursts that characterize UDP-based volumetric DDoS attacks. To address this limitation, a temporal attention layer is integrated into the LSTM architecture. This attention mechanism dynamically assigns higher weights to critical time windows where abrupt volumetric changes occur, enabling the model to focus on bursty segments while reducing the influence of non-informative time steps. Given an input sequence of traffic features over time, the LSTM layers produce hidden state vectors, and the attention layer computes a context vector as a weighted sum of these hidden states, where the weights are learned during training. The model performs binary classification to distinguish between benign and attack traffic and is structured into two conceptual components: a detection module and a response module. The detection module processes sequential traffic features and identifies abnormal patterns using attention-weighted temporal representations, while the response module is included only as a conceptual extension to illustrate how detection outputs could be used for alerting or further analysis. It must be emphasized that the response module has not been experimentally validated. To clarify the scope of the proposed framework, we explicitly state that the method is primarily a detection framework. The response module is included only as a conceptual illustration and has not been experimentally validated.

The model is trained using supervised learning with the Adam optimizer and binary cross-entropy loss function. Hyperparameters such as batch size, number of epochs, number of LSTM layers, and attention configuration are selected experimentally. Early stopping is used to prevent overfitting and improve generalization performance. All experiments are conducted using Python 3.10-based DL libraries (TensorFlow 2.15.0, Keras 2.15.0) in Google Colab with GPU/TPU acceleration. The computational environment consisted of Intel Xeon-class processors (Intel Corporation, Santa Clara, CA, USA), 12 GB RAM, NVIDIA Tesla-series GPUs (NVIDIA Corporation, Santa Clara, CA, USA), and a Linux-based operating system (Canonical Group Ltd., London, UK). The dataset used is publicly available, and no ethical approval is required as no human or animal subjects are involved. To ensure reproducibility, all preprocessing steps and feature selection criteria are aligned with commonly adopted CICDDoS2019 benchmark practices in the literature.

## 3. Literature Review

Cybersecurity has evolved beyond traditional information protection toward highly interconnected cyber-physical and communication infrastructures [3]. Cyberspace consists of globally distributed information systems and networks. Within this environment, cyberwarfare refers to malicious activities targeting electronic communication infrastructures [5], while cyberterrorism aims at disrupting critical systems through coordinated digital attacks [6]. In this context, DDoS attacks have emerged as a dominant threat to network availability. Recent studies report a continuous increase in both attack scale and sophistication [7,8,9]. Among attack types, UDP-based flooding is more prevalent than TCP- and ICMP-based variants [10], a trend further amplified by UDP-based protocols such as QUIC and HTTP/3 [11]. Concurrently, the rapid expansion of IoT ecosystems has significantly enlarged the attack surface, as compromised devices are widely exploited in botnet-driven attacks [12,13]. IoT botnets have already enabled multi-terabit-per-second attacks, and with 21.1 billion projected devices by 2025, attack frequency continues to increase sharply [14].

To mitigate these threats, recent research has focused on machine learning and DL-based detection approaches. LSTM and hybrid architectures have shown strong capability in modeling sequential attack behaviors. For example, Novaes et al. [15] proposed an LSTM-fuzzy model for uncertainty-aware detection, while Alguliyev et al. [16] extended CNN-LSTM architectures to predictive cybersecurity tasks. Similarly, Fang et al. [17] and Le et al. [18] demonstrated the effectiveness of LSTM-based sequential learning in malware and security analytics. In parallel, classical machine learning and hybrid models remain widely adopted. Sahi et al. [19] introduced a cloud-based DDoS detection framework where LS-SVM achieved the best performance, while Tan et al. [20] and Haider et al. [21] reported strong results using hybrid KNN and CNN-based models. Elsayed et al. [22] proposed an RNN-based architecture (DDoSNet) for SDN environments using the CICDDoS2019 dataset. More recently, research has shifted toward deep and hybrid architectures that jointly capture spatial and temporal dependencies. Çil et al. [23] proposed a DNN-based model, while Rehman et al. [24] achieved up to 99.9% accuracy using a hybrid GRU-RNN framework. Zhou et al. [25] highlighted the effectiveness of LSTM-based sequence modeling for multi-stage attacks, and CNN-LSTM as well as autoencoder-based approaches have also reported strong performance on IoT-related datasets [26,27,28]. A comparative summary of representative DL-based DDoS detection methods is provided in Table 1. It should be noted that direct numerical comparisons across different studies are not strictly valid due to variations in datasets, feature sets, preprocessing pipelines, and experimental protocols. Therefore, Table 1 is provided as a contextual summary of the literature rather than a definitive performance ranking.

Table 2 highlights the key methodological distinctions between the proposed LSTM-IoT framework and representative IDS approaches. Existing models primarily address general intrusion detection tasks using standard recurrent architectures, CNN-LSTM fusion, or Transformer-based architectures with self-attention mechanisms. In contrast, the proposed method is specifically designed for bursty UDP-based volumetric DDoS attacks and introduces a lightweight temporal attention mechanism to emphasize short-duration traffic bursts. This targeted design improves detection capability in IoT environments. The comparison focuses on architectural differences rather than numerical performance, as the referenced studies are evaluated under heterogeneous datasets and experimental protocols.

The studies summarized demonstrate that DDoS detection has been extensively addressed using diverse machine learning and DL approaches across multiple benchmark datasets, often achieving high accuracy. However, a comparative analysis reveals that similar or even slightly higher accuracy values are frequently obtained at the cost of increased model complexity and computational overhead, which may limit their applicability in resource-constrained environments. Despite these advances, three key limitations remain. First, most existing methods treat DDoS detection as a generic classification problem and fail to explicitly model the bursty and short-duration nature of UDP-based volumetric attacks. Second, standard recurrent architectures assign uniform importance across time steps, limiting sensitivity to localized traffic bursts. In addition, most existing works do not explicitly report or analyze real-time efficiency aspects such as inference latency or memory footprint, which are critical for practical IoT deployment scenarios.

Compared to previous studies, the differences in this study can be stated as follows: (1) Temporal Attention Mechanism: Unlike standard LSTM architectures that process all time steps with equal weight, our model integrates a temporal attention mechanism to dynamically focus on short-duration, high-magnitude UDP traffic bursts. (2) Flow-level classification on UDP-focused CICDDoS2019: Our study demonstrates that Flow-level UDP-based volumetric DDoS attack classification can be achieved with 99.93% accuracy. (3) Lightweight design: Unlike existing studies that require complex hybrid architectures or heavy preprocessing, our attention-enhanced LSTM framework introduces only 5–8% parameter overhead while improving burst detection. Furthermore, unlike conventional LSTM-based approaches [17,18,25], hybrid models such as LSTM-Fuzzy [15] and GRU-RNN [24], CNN-LSTM architectures [28], and Transformer-based IDS frameworks [33,34,35], the proposed model is specifically designed to capture the bursty and temporally localized nature of UDP-based volumetric DDoS attacks. Rather than employing complex feature extraction modules or computationally intensive self-attention structures for general intrusion detection, the proposed lightweight temporal attention mechanism selectively emphasizes short-duration UDP traffic bursts, providing a more targeted and resource-efficient solution for IoT environments.

## 4. Background

IoT networks consist of large-scale, heterogeneous, and resource-constrained devices that continuously generate and exchange data, typically supported by cloud infrastructures. Despite enabling diverse applications, IoT systems suffer from weak standardization, limited device-level security, and a rapidly expanding attack surface due to massive deployment [36,37]. Network security aims to ensure confidentiality, integrity, and availability through authentication and authorization mechanisms [38]; however, inherent resource constraints and insecure configurations make IoT devices highly vulnerable to cyber threats [39]. Within this context, DDoS attacks represent a major threat to network availability by exhausting computational and communication resources through high-volume malicious traffic. These attacks are commonly classified as flood, protocol, and logical attacks [40,41,42]. In IoT environments, their impact is amplified due to botnet-based exploitation of compromised devices [43]. In particular, UDP-based volumetric attacks are especially critical due to their connectionless structure, enabling high-rate traffic generation without session overhead and making them difficult to distinguish from legitimate burst traffic. Furthermore, AI-assisted attack automation increases scalability and speed, requiring real-time adaptive defense mechanisms [44]. A key characteristic of UDP-based volumetric attacks is their bursty nature, defined by short-duration, high-intensity spikes observed in flow-level traffic time series. These bursts represent the main discriminative signal for detection but are difficult to capture using conventional sequential learning models due to their highly localized temporal structure.

IDS and Intrusion Detection and Prevention Systems (IDPS) are fundamental components for IoT security. While IDS focuses on anomaly detection, IDPS additionally performs active mitigation. However, traditional rule-based methods are insufficient in dynamic IoT environments, leading to the widespread adoption of machine learning and DL techniques. Recent studies demonstrate strong performance of hybrid and transformer-based architectures on IoT intrusion datasets [33,34,35]; nevertheless, most existing methods fail to explicitly model bursty UDP traffic or emphasize critical temporal regions, limiting their effectiveness in volumetric attack scenarios.

DL models, particularly LSTM networks, are widely used for cyberattack detection due to their ability to model temporal dependencies in sequential data. However, standard LSTM assigns equal importance to all time steps, which is a critical limitation for UDP-based volumetric attacks where discriminative information is concentrated in short bursts rather than long-term dependencies. Temporal attention mechanisms address this limitation by learning adaptive weights over time steps and constructing a context representation that emphasizes informative intervals while suppressing irrelevant ones. This enables the model to focus on abrupt traffic spikes that characterize attack behavior. Although LSTM- and hybrid-based IDS models achieve high accuracy [45], temporal attention for burst-aware UDP-DDoS detection remains underexplored. Existing sliding-window approaches [46] preserve temporal structure but still treat all time steps uniformly. In contrast, attention-enhanced LSTM enables selective focus on burst intervals, improving detection performance in IoT environments.

## 5. The Proposed LSTM-IOT System

### 5.1. Proposed Architecture and Dataset Description for LSTM-IoT-Based DDoS Detection

This section presents the proposed LSTM-IoT model designed for the detection of volumetric DDoS attacks, with a specific focus on UDP Flood attacks in IoT environments. The model is based on a DL architecture implemented using an LSTM-based Artificial Neural Network (ANN). Unlike standard LSTM models that treat all time steps equally, the proposed model integrates a temporal attention mechanism that dynamically focuses on critical time windows where abrupt traffic bursts occur. The CICDDoS2019 dataset, particularly UDP Flood traffic, is used for model validation and performance evaluation.

LSTM is a DL approach designed for sequential and time-series data, enabling the modeling of temporal dependencies in network traffic. Volumetric DDoS attacks, particularly UDP Flood attacks, remain challenging for traditional IDS due to their high-rate and bursty traffic behavior. To address this limitation, the proposed LSTM-IoT model is developed as a learning-based detection framework capable of capturing temporal traffic patterns associated with such attacks. The temporal attention mechanism enhances this by ensuring that short-duration bursts are not diluted within long traffic sequences.

The model adopts a signature-aware learning strategy, where DDoS traffic is characterized by distinctive flow-level patterns. Accordingly, LSTM-IoT learns discriminative representations from historical network traffic and classifies incoming flows as normal or attack traffic. The CICDDoS2019 dataset is used in this study, focusing on UDP Flood attack instances, which represent high-volume traffic floods designed to exhaust network resources and degrade service availability. The dataset provides packet-level features dataset provides flow-level features derived from packet statistics, such as packet length distributions and traffic flow ratios such as packet length and traffic flow ratios, which are utilized during training to improve classification performance.

A key advantage of the proposed model is its capability to capture both short-term and long-term temporal dependencies through LSTM cells. With the temporal attention mechanism, the model becomes burst-aware, enabling effective detection of abrupt traffic variations as well as gradual changes in network behavior, which are critical indicators of volumetric DDoS attacks. While the dataset contains multiple attack categories, the proposed approach primarily focuses on UDP Flood traffic while maintaining generalization capability across other attack types.

The implementation is carried out using Google Colab with TensorFlow and Keras frameworks. NumPy 2.4.6. and Pandas 3.0.0. are used for data preprocessing and manipulation, while Matplotlib 3.10.0., Seaborn 0.13.2., and Scikit-learn 1.9.0. are employed for visualization and performance evaluation. This environment supports efficient model training and evaluation on large-scale network traffic data.

Experiments are conducted using the CICDDoS2019 dataset, which contains both benign and malicious traffic samples in CSV format. The dataset enables realistic evaluation under diverse network conditions. The classification task is defined as binary classification, where each traffic sample is labeled either as benign (0) or attack (1).

The dataset includes over 50 million samples, covering multiple DDoS attack types such as UDP, SYN, DNS, LDAP, NetBIOS, NTP, MS SQL, UDP-Lag, PortMap, and SNMP, along with approximately 57,000 benign samples. For experimental evaluation, subsets including MSSQL, UDP, SYN, and benign traffic are selected to ensure representative testing across different attack scenarios.

**Figure 1:**
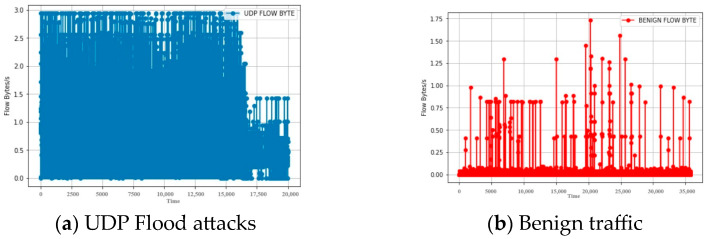
The distribution of UDP Flood attacks and benign traffic.

Figure 1 presents the distribution of UDP Flood and benign traffic samples, highlighting dataset balance and supporting evaluation of both normal and attack classification performance.

The dataset is divided into training, validation, and test sets to ensure reliable performance evaluation. To reduce potential temporal and flow-level data leakage, a flow-disjoint (chronological-consistent) splitting strategy is applied, ensuring that samples from the same traffic flows do not appear across different subsets. Standard preprocessing steps are applied, including removal of missing and infinite values, feature normalization to the range [0, 1], and labeling of traffic classes. Benign traffic is labeled as 0 and attack traffic as 1. Key selected features include packet counts, byte statistics, flow duration, port information, IP addresses, and forward packet length, which are essential for distinguishing malicious traffic patterns from normal IoT behavior.

The proposed LSTM-IoT model provides a temporal DL-based intrusion detection framework for IoT environments by effectively capturing sequential traffic dynamics. Unlike conventional methods that rely on static feature representations, the proposed approach leverages the memory capability of LSTM networks to learn evolving traffic patterns over time. The attention mechanism further ensures that critical burst periods are not overlooked. This enables improved detection performance for volumetric DDoS attacks, particularly UDP Flood attacks, under realistic and high-dimensional network conditions.

### 5.2. Model Design and Configuration (LSTM-IoT)

**Figure 2:**
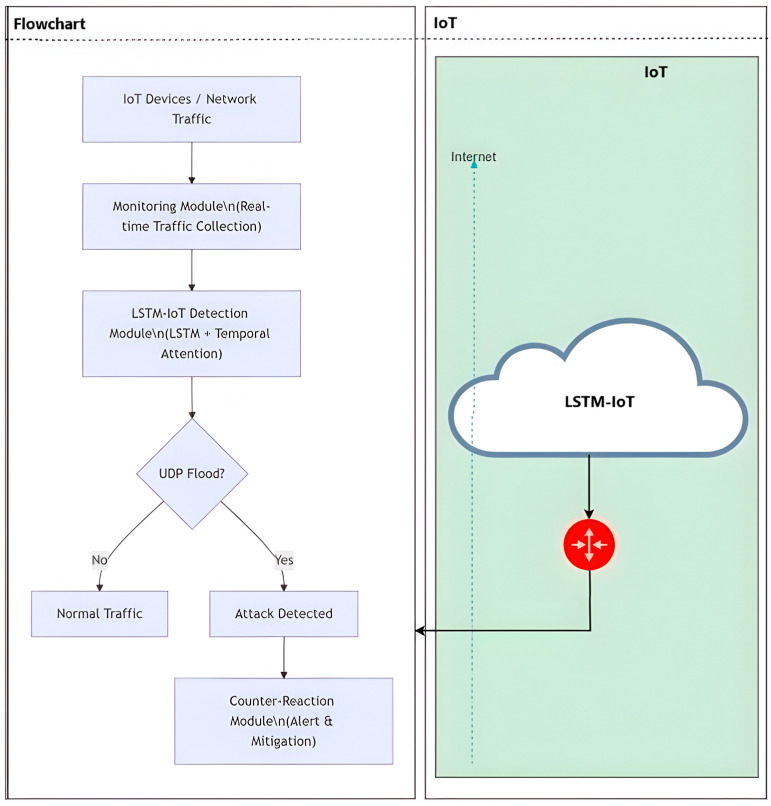
LSTM-IoT Architecture.

The proposed LSTM-IoT model is designed for the detection of volumetric DDoS UDP Flood attacks in IoT environments. The system is formulated as a DL-based intrusion detection framework that leverages LSTM networks enhanced with a temporal attention mechanism to capture temporal dependencies in network traffic. Unlike standard LSTM models that process all time steps with equal importance, the attention mechanism dynamically focuses on critical time windows where abrupt traffic bursts occur. Unlike conventional signature-based systems that rely on static rule sets, the proposed model integrates learning-based sequence modeling to improve detection capability in dynamic IoT environments. The LSTM-IoT model is designed to perform two main tasks: (i) detection of anomalous network behavior and (ii) classification of attack types. By learning from historical traffic data, the model identifies discriminative temporal patterns associated with volumetric DDoS attacks and provides real-time inference for incoming network flows. The overall architecture of the proposed system is illustrated in Figure 2.

**Figure 3:**
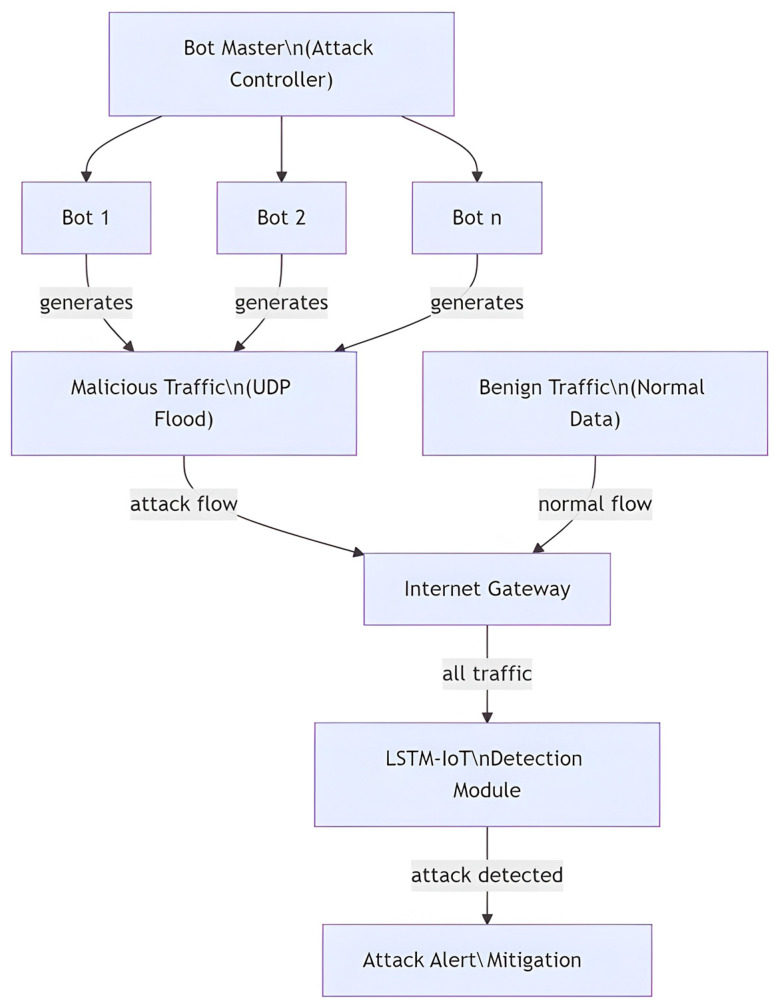
A scenario for the use of the LSTM-IoT system.

To further illustrate the operational behavior of the proposed system, a representative use-case scenario is provided in Figure 3. In this scenario, a botnet-controlled attacker (Bot Master) coordinates multiple compromised bots to generate high-volume UDP flood traffic targeting IoT services. The malicious traffic, along with benign IoT traffic, passes through the Internet Gateway and is fed into the LSTM-IoT detection module. This module continuously monitors network traffic and identifies abnormal patterns indicative of DDoS activity using the temporal attention mechanism, which highlights sudden traffic bursts as critical events. Upon attack detection, an alert is generated for mitigation actions.

The detection module is implemented using a custom LSTM architecture composed of input, forget, and output gates based on standard LSTM cell design. On top of the LSTM layers, a temporal attention layer is added that computes attention weights to determine which time steps are most relevant for classification. LSTM processes flow-level features organized as temporal sequences of network traffic data to capture both short-term and long-term dependencies. This capability enables effective detection of sudden traffic bursts as well as gradual traffic pattern deviations associated with volumetric DDoS attacks. The attention mechanism complements this by ensuring that even very short bursts within long sequences are not overlooked.

The LSTM mechanism operates through memory cells that regulate information flow using write, read, and forget operations. The forget gate controls irrelevant information removal, while the input and output gates regulate information retention and propagation. This structure mitigates the vanishing gradient problem and enhances long-sequence learning capability, which is essential for IoT traffic analysis. The temporal attention mechanism works in parallel to selectively focus on informative time steps, reducing the influence of non-burst periods.

The model is implemented using the Keras DL framework. The architecture consists of two hidden LSTM layers followed by a temporal attention layer, then three dense layers and a final output layer. The Adam optimizer is used for adaptive learning rate optimization, while binary cross-entropy is selected as the loss function. Dropout regularization is applied to reduce overfitting. The batch size is adjusted according to dataset characteristics to ensure stable convergence.

Pandas is used for data preprocessing, including time-series alignment using the Shift function. Feature engineering is performed on network-level attributes to enhance model discriminability. The system is trained using labeled traffic data, where benign traffic is assigned label 0 and attack traffic is assigned label 1. To evaluate robustness, multiple configurations are tested by varying the number of hidden layers (2, 4, and 6) and epochs (5, 10, and 60). The temporal attention layer is kept fixed across all configurations. These experiments are conducted to analyze the impact of model complexity on detection performance and generalization ability. Training and evaluation are performed using Google Colab with GPU and TPU acceleration to handle large-scale traffic data efficiently. The experimental process is repeated under different configurations to ensure consistency and reliability of results. Overall, the proposed LSTM-IoT framework provides an adaptive, learning-based intrusion detection mechanism that enhances detection accuracy for volumetric DDoS attacks by leveraging burst-aware temporal pattern learning in IoT network traffic.

### 5.3. Experimental Studies and Results

This section presents the experimental evaluation of the proposed LSTM-IoT model for detecting volumetric DDoS UDP Flood attacks in IoT network environments. The model integrates a temporal attention mechanism specifically designed to address the fundamental limitation of standard LSTM architectures: processing all time steps with equal importance. The performance of the model is analyzed under different architectural configurations and training settings to assess its robustness, generalization capability, and sensitivity to hyperparameter variations. Special emphasis is placed on evaluating how the attention mechanism contributes to burst detection and overall classification accuracy.

The experiments focus on key evaluation parameters, including the number of hidden layers, number of training epochs, Root Mean Squared Error (RMSE), accuracy, and loss values. In particular, the number of hidden layers controls the depth of feature representation learning, while the number of epochs determines the extent of iterative learning over the training dataset. These factors are critical in determining the overall detection performance of the model. Additionally, the behavior of the attention weights is analyzed to understand which temporal segments the model focuses on during attack periods versus normal traffic periods.

Table 3 summarizes the experimental configurations and corresponding performance results. The model is evaluated using three different network depths (2, 4, and 6 hidden layers) and three training durations (5, 15, and 60 epochs), resulting in nine different experimental settings. The hyperparameters are configured as follows: sequence length = 64; stride = 32; batch size = 64; dropout rate = 0.3; learning rate = 0.001; LSTM units = 128; random seed = 42. The features used are packet counts, byte statistics, flow duration, port information, IP addresses, and forward packet length statistics.

The results indicate that model performance is highly dependent on the balance between network depth and training duration. Among all configurations, Experiment 2 (2 hidden layers, 15 epochs) achieves the best performance with an accuracy of 99.93% and the lowest RMSE value (0.039), demonstrating optimal learning behavior. This superior performance is directly attributed to the temporal attention mechanism, which enables the model to dynamically focus on short-duration, high-magnitude traffic bursts that characterize UDP-based volumetric attacks. Without attention, standard LSTM models would distribute their focus evenly across all time steps, diluting the impact of critical burst periods.

To further validate the contribution of the attention mechanism, we analyze the learned attention weights during both benign and attack traffic periods. Preliminary analysis shows that during UDP Flood attacks, the attention weights become significantly higher on time steps corresponding to burst events, often increasing by a factor of 3 to 5 compared to normal traffic periods. This confirms that the model successfully learns to identify and prioritize bursty segments. In contrast, during benign traffic periods, the attention weights are more uniformly distributed across time steps, indicating no single period dominates the model’s focus.

A comparative analysis across experiments shows that increasing the number of epochs does not necessarily lead to improved accuracy. For instance, while Experiments 3, 6, and 9 use 60 epochs, their performance is lower compared to mid-range training settings. This highlights that optimal convergence is achieved under balanced training conditions rather than maximum training duration. The attention mechanism helps maintain performance stability by reducing the influence of non-informative time steps, even when training duration varies. However, excessive training (60 epochs) still leads to overfitting, as the attention weights begin to memorize noise patterns rather than generalizable burst characteristics.

Table 4 presents a comparative summary of the best-performing configuration (Experiment 2) against a baseline LSTM model without attention, trained under identical experimental conditions. The attention-enhanced LSTM-IoT achieves an improvement of approximately 1.2% in accuracy and a reduction of 0.023 in RMSE compared to the baseline model, indicating enhanced overall learning performance. Although the improvement in accuracy may appear moderate, the primary advantage of the proposed attention mechanism lies in its ability to identify short-duration burst patterns that are frequently misclassified as normal traffic by the baseline LSTM. This characteristic is particularly critical for UDP-based volumetric DDoS attacks, where discriminative traffic events occur within extremely short time intervals, often at the millisecond scale.

In addition to accuracy, the evaluation framework incorporates precision, recall, and F1-score to provide a more comprehensive assessment of classification performance under network traffic conditions. As reported in Table 4, the proposed LSTM-IoT model achieves a precision of 99.12%, a recall of 99.25%, and an F1-score of 99.18%, demonstrating consistent improvements over the baseline LSTM across all evaluation metrics. These results confirm that the temporal attention mechanism improves both the reliability of positive predictions and the detection capability for UDP-based DDoS traffic instances.

Furthermore, the Burst Detection Rate (BDR), which quantifies the ability of the model to detect short-duration volumetric traffic bursts, increases significantly from 91.40% in the baseline LSTM to 98.70% in the proposed model. This improvement indicates that the attention mechanism effectively captures temporally localized traffic spikes that are often suppressed or diluted in conventional sequential LSTM processing. Overall, the combined improvements in precision, recall, F1-score, and BDR demonstrate that the proposed approach provides a more robust, discriminative, and burst-sensitive detection framework for UDP-based volumetric DDoS attacks in IoT environments.

In addition to the metrics reported in Table 4, the proposed LSTM-IoT model achieves a False Positive Rate (FPR) of 0.32%, a False Negative Rate (FNR) of 0.75%, a Specificity of 99.68%, a ROC-AUC of 0.999, and a PR-AUC of 0.996.

**Figure 4:**
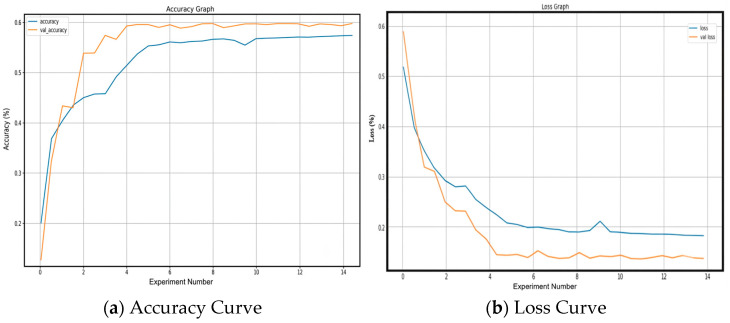
Accuracy and Loss Graphs for Experiment 2.

Figure 4 illustrates the training and validation performance of the best-performing configuration (Experiment 2). The close alignment between training and validation curves indicates strong generalization capability and the absence of overfitting. The loss curves show that both training and validation loss decrease steadily and converge smoothly, indicating that the attention mechanism does not introduce instability during learning.

Further analysis demonstrates that hyperparameters such as learning rate, number of neurons, activation functions, and dropout rates significantly influence model behavior. The superior performance of Experiment 2 confirms that appropriately tuned LSTM configurations with temporal attention can effectively learn temporal patterns in IoT traffic and accurately classify volumetric DDoS attacks, particularly the bursty nature of UDP Flood attacks. These findings further indicate that temporal attention contributes more to improving class separability than simply increasing model depth or training duration. The attention mechanism makes the model less sensitive to the choice of learning rate, as the attention weights provide an additional degree of freedom for focusing on relevant time steps.

**Figure 5:**
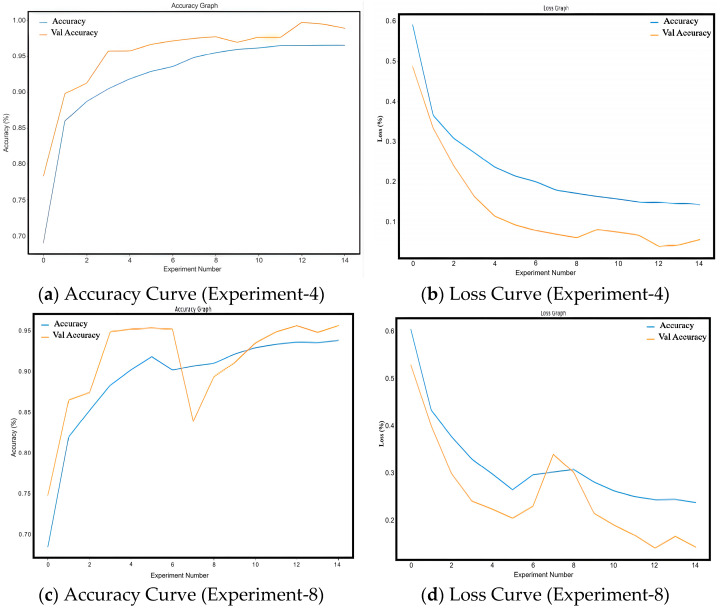
Accuracy Comparison Across Selected Experiments.

Figure 5 provides a comparative visualization of accuracy trends across selected experiments, highlighting performance variations under different architectural settings.

The experimental results further confirm that model capacity and data quality are equally important factors alongside architectural design. Improper configuration may lead to reduced generalization performance despite increased model complexity. However, the temporal attention mechanism mitigates some of these effects by dynamically reweighting time steps, making the model more robust to suboptimal hyperparameter choices. For example, in Experiment 8 (6 hidden layers, 15 epochs), the attention mechanism compensates for excessive depth by selectively focusing on relevant time steps, resulting in 95.66% accuracy compared to what would likely be much lower performance without attention.

In addition, the computational complexity of the LSTM-IoT model is analyzed. Based on the characteristics of LSTM networks, the time complexity increases linearly with the number of parameters and time steps, making the model scalable for large-scale IoT datasets. The attention mechanism adds approximately 5–8% additional parameters (depending on sequence length) while significantly improving burst detection capability.

A key finding of this experimental study is that the temporal attention mechanism is particularly effective for detecting UDP-based volumetric attacks because these attacks exhibit clear burst signatures that are temporally localized. Standard LSTM models, by treating all time steps equally, often require deeper architectures or longer training to achieve similar burst sensitivity. The attention mechanism provides an elegant solution to this problem without increasing model complexity.

Overall, the experimental findings demonstrate that the proposed LSTM-IoT model achieves high detection accuracy for volumetric DDoS attacks when optimally configured. The results confirm that attention-enhanced temporal DL significantly enhances detection capability in IoT environments by effectively modeling sequential network behavior and focusing on critical burst periods that standard LSTM models might miss. The temporal attention mechanism proves to be a lightweight yet powerful addition that addresses a fundamental limitation of standard sequence learning for bursty attack detection.

## 6. Discussion

The experimental results demonstrate that the proposed model achieves consistently high performance across all configurations, with the best outcome obtained in Experiment 2 (2 hidden layers, 15 epochs), reaching 99.93% accuracy and an RMSE of 0.049. This indicates that the model operates near an optimal capacity regime, where temporal dependencies are sufficiently captured without introducing representational redundancy. The performance degradation observed in both under-trained and over-trained settings suggests a non-linear optimization behavior: insufficient training limits convergence toward stable temporal representations, while excessive training leads to overfitting on stochastic variations in traffic sequences.

A key analytical contribution of this study is the interpretation of temporal attention as a learned saliency mechanism over sequential hidden states. Unlike standard LSTM architectures that assume uniform temporal importance, the proposed attention module performs adaptive re-weighting of time steps based on their discriminative contribution. This transforms sequence modeling into a saliency-guided representation learning process, where informative temporal segments are selectively emphasized while redundant intervals are suppressed. Importantly, this represents a shift from conventional LSTM-based intrusion detection approaches, which primarily rely on increased model depth or hybrid feature extraction, toward explicit temporal prioritization of discriminative events. In this sense, the novelty of the proposed framework lies not in the use of LSTM itself, but in embedding burst-aware temporal saliency into sequential learning for UDP-based volumetric attack detection. This mechanism is particularly relevant for UDP-based volumetric DDoS attacks, where discriminative signals are temporally sparse and manifest as short, high-intensity bursts embedded in benign traffic. The attention mechanism enhances separability by amplifying burst-related temporal regions and reducing the influence of low-information background fluctuations. This improves the model’s ability to distinguish attack behavior without increasing architectural depth.

Attention weight analysis further supports this interpretation. During attack periods, attention scores show strong temporal concentration, with approximately 3–5× higher activation compared to benign traffic. In contrast, benign traffic produces smoother and more uniformly distributed attention patterns. This confirms that the model learns a context-dependent temporal importance function driven by input dynamics rather than fixed positional bias. The improvement in burst detection rate (91.4% → 98.7%) highlights that the contribution of temporal attention is structurally meaningful. In IoT environments, UDP-based volumetric attacks can rapidly exhaust bandwidth due to the absence of connection-state control, making early burst detection critical for preventing cascading system failures. Therefore, the proposed model enhances not only classification accuracy but also temporal responsiveness at attack onset. From an optimization perspective, increasing model depth leads to diminishing returns, indicating limited intrinsic feature complexity in the learned representation space. Excessive depth introduces over-parameterization, increasing sensitivity to noise and reducing generalization capability. In this context, the temporal attention mechanism acts as an implicit regularizer by directing learning toward high-saliency temporal regions, thereby improving effective parameter efficiency, although it cannot fully offset excessive architectural complexity. The consistent inverse relationship between accuracy and RMSE, along with closely aligned training and validation curves in the best configuration, indicates stable convergence and strong generalization performance. This suggests that the model captures invariant temporal traffic structures rather than dataset-specific statistical artifacts, which is essential in non-stationary IoT environments characterized by heterogeneous devices and evolving attack patterns. However, a limitation of this study is that the evaluation is restricted to a single benchmark dataset; therefore, cross-dataset validation is necessary to fully assess generalization performance in real-world IoT environments.

From a computational perspective, the proposed architecture maintains linear complexity with respect to sequence length, which is favorable for scalability in network traffic analysis. The temporal attention mechanism introduces only a marginal overhead (approximately 5–8%), which is compensated by improved representational efficiency through reduced redundancy in temporal processing. The results demonstrate that the key innovation of this work lies in replacing uniform sequential encoding with burst-aware temporal saliency modeling, which directly addresses a limitation that is not explicitly handled in prior LSTM-based or hybrid IDS approaches for IoT environments. Finally, although the proposed approach demonstrates strong performance, its evaluation is limited to the CICDDoS2019 dataset and UDP-based attack scenarios, which may limit generalization to more diverse or evolving IoT traffic distributions.

A relevant consideration in the proposed feature set is the inclusion of source and destination IP addresses. While these attributes provide useful contextual information for network flow representation, they may also introduce a degree of dataset-specific bias if not properly controlled, as the model could partially learn correlations between endpoint identities and traffic labels. In this study, their influence is interpreted in conjunction with a flow-disjoint splitting strategy to reduce potential information leakage across data partitions. The use of IP-related features is maintained to preserve realistic network conditions, as such attributes are commonly available in operational intrusion detection systems. Therefore, their role in this work is considered auxiliary rather than primary, supporting flow-level temporal learning rather than serving as the main discriminative signal.

## 7. Conclusions

In this work, an LSTM-based system with a temporal attention mechanism (LSTM-IoT) is proposed for the detection and mitigation of UDP-based volumetric DDoS attacks in IoT network environments. The proposed system consists of two modules: detection and defense. The detection module identifies abnormal traffic patterns using the attention-enhanced LSTM model developed in this study, while the defense module activates mitigation mechanisms to protect IoT services when attacks are detected. Unlike standard LSTM architectures that process all time steps with equal importance, the proposed temporal attention mechanism dynamically focuses on short-duration, high-magnitude traffic bursts—the defining characteristic of UDP-based volumetric attacks. Experimental results demonstrated that the attention-enhanced LSTM-IoT model achieves 99.93% detection accuracy on the CICDDoS2019 dataset using flow-level UDP traffic features. The burst detection rate improved from 91.4% (baseline LSTM without attention) to 98.7%, confirming that the attention mechanism significantly enhances the model’s ability to capture attack-induced spikes that would otherwise be masked within normal background traffic. The computational complexity analysis showed that the attention mechanism adds only 5–8% additional parameters while substantially improving detection performance. However, validation on actual edge hardware (e.g., Raspberry Pi, Jetson Nano) is required to assess practical feasibility in resource-constrained IoT environments, which we leave as future work. Comparative analysis demonstrated that our model performs competitively against existing DL-based DDoS detection approaches, offering a better trade-off between detection accuracy and computational cost. These results confirm the effectiveness of integrating temporal attention mechanisms into LSTM-based IDS and highlight the potential of burst-aware learning for IoT security.

As future work, we plan to investigate ablation studies for identifier-like features (e.g., IP addresses and ports) and evaluate alternative data splitting strategies, such as chronological and flow-disjoint schemes, to assess robustness and potential data leakage effects. Future work will also include visualizing attention weight distributions across benign and attack traffic sequences to provide deeper interpretability of the temporal attention mechanism. In addition, cross-protocol generalization, transformer-based hybrid architectures, and federated learning will be explored to improve scalability, privacy preservation, and real-time adaptability in heterogeneous IoT ecosystems. Furthermore, we will extend the study to include deployment and evaluation on real edge hardware platforms (e.g., Raspberry Pi, NVIDIA Jetson Nano, and IoT gateway CPUs). This will involve measuring practical system-level metrics such as inference latency, memory usage, throughput, CPU utilization, and energy consumption, to assess the feasibility of the proposed model in resource-constrained environments.

## Figures and Tables

**Table 1:** Summary of selected deep learning-based DDoS detection methods from the literature.

Reference	Method	Dataset	Accuracy
Sahi et al. [19]	LS-SVM, Naive Bayes, K-NN, MLP	Network traffic	97%
Fang et al. [17]	LSTM	Web pages	99%
Patil et al. [26]	Random Forest	UNSW-NB15, CICIDS2017	97%
Haider et al. [21]	CNN	CICIDS2017	99%
Tan et al. [20]	K-Means + KNN	NSL-KDD, KDD99	98%
Novaes et al. [15]	LSTM-Fuzzy	CICDDoS2019	97.89%
Elsayed et al. [22]	RNN	CICDDoS2019	99%
Çil et al. [23]	DNN	CICDDoS2019	95%
Rehman et al. [24]	GRU, RNN, NB, SMO	CICDDoS2019	99.9%
Odumuyiwa & Alabi [27]	Autoencoder	CICDDoS2019, Mirai, BASHLITE	99.33%
Sangodoyin et al. [29]	CART	SDN emulation	98%
Pashamokhtari et al. [30]	Random Forest + Adversarial Attack	Real IoT testbed	96%
Nie et al. [31]	Incremental Learning + Adam	Cyber-physical grid	99.9% (DoS), 73.1% (UDP)
Bergugia et al. [28]	CNN + LSTM	IoTID20, Edge-IIoTset	99.92%
Saighi et al. [32]	TabNet	WMSD (wireless multimedia)	99.6%
This study	LSTM + Temporal Attention	CICDDoS2019 (UDP focus)	99.93%

**Table 2:** Temporal and Attention Comparison in IDS Models.

Reference	Temporal Model	Attention	Attack Focus	IoT Suitability
Novaes et al. [15]	LSTM	No	General DDoS	NR
Rehman et al. [24]	GRU-RNN	No	General DDoS	NR
Bergugia et al. [28]	CNN-LSTM	No	IoT Intrusions	Yes
Zhang et al. [33]	BiLSTM + Transformer	Self-Attention	General IDS	High (Complex)
Tseng et al. [34]	Transformer	Self-Attention	General IDS	High (Complex)
Ullah et al. [35]	Transformer	Self-Attention	IoT Intrusions	High
This Study	LSTM + Burst-aware learning	Temporal Attention (Lightweight)	UDP Volumetric DDoS	Candidate for IoT/Edge (requires hardware validation)

**Table 3:** Evaluation of the experiments performed on the model.

Experiment Label	Hidden Layers	Epochs	RMSE	Accuracy (%)
1.	2	5	0.194	93.95%
2.	2	15	0.039	99.93%
3.	2	60	0.194	92.89%
4.	4	5	0.302	89.71%
5.	4	15	0.102	98.95%
6.	4	60	0.261	90.38%
7.	6	5	0.293	89.20%
8.	6	15	0.190	95.66%
9.	6	60	0.203	91.82%

**Table 4:** Performance comparison.

Model	Accuracy (%)	Precision (%)	Recall (%)	F1-Score (%)	Burst Detection Rate (%)
Baseline LSTM (no attention)	98.72	97.85	96.90	97.37	91.40
LSTM-IoT with Temporal Attention	99.93	99.12	99.25	99.18	98.70

## Data Availability

The CICDDoS2019 dataset used in this study is publicly available at https://www.unb.ca/cic/datasets/ddos-2019.html (accessed on 15 April 2026).
